# The Experimental Study of Periodontal Ligament Stem Cells Derived Exosomes with Hydrogel Accelerating Bone Regeneration on Alveolar Bone Defect

**DOI:** 10.3390/pharmaceutics14102189

**Published:** 2022-10-14

**Authors:** Yang Zhao, Yujia Gong, Xianbo Liu, Jia He, Bowen Zheng, Yi Liu

**Affiliations:** Department of Orthodontics, School and Hospital of Stomatology, China Medical University, Liaoning Provincial Key Laboratory of Oral Diseases, Shenyang 110002, China

**Keywords:** periodontal membrane stem cells, exosome, hydrogel, alveolar bone defect repair, bone regeneration

## Abstract

Introduction: this study was conducted to investigate the osteogenic ability of periodontal ligament stem cells (PDLSCs) derived exosomes (PDLSCs-Exos) and the effect of PDLSCs-Exos with hydrogel on alveolar bone defect repairment in the rat. Methods: the PDLSCs were obtained through primary cell culture, and PDLSCs-Exos were purified by the ultracentrifugation method. The CCK-8 kit and ALP staining were used to explore the effect of PDLSCs-Exos on promoting the proliferation and osteogenic differentiation of bone marrow mesenchymal stem cells (BMSCs). In vivo, the alveolar bone defect models were made mesial to the bilateral maxillary first molars of rats. MicroCT, HE staining, and Masson staining were used to analyze the new bone at the bone defect of rats. Results: the periodontal ligament stem cells and the periodontal ligament stem cells derived exosomes were successfully extracted. The results of the CCK-8 kit and ALP staining showed PDLSCs-Exos significantly promoted the proliferation osteogenic differentiation of BMSCs. In vivo experiment results revealed that compared with the control group and the hydrogel group, the rats in the hydrogel with exosomes group showed more new bone formation in alveolar bone defects. Conclusion: Periodontal ligament stem cells and exosomes derived from periodontal ligament stem cells were successfully extracted. The results demonstrated that the hydrogel successfully delivered periodontal ligament stem cells derived exosomes for repairing alveolar bone defects in rats in vivo at the initial stage.

## 1. Introduction

The alveolar bone defect has aroused widespread concern worldwide. The alveolar bone defect may not only lead to tooth loss but also affect mouth functioning and facial aesthetics. At present, the main approaches to treating alveolar bone tissue defects are auto-transplantation and allotransplantation, but these approaches have many limitations. For example, auto-transplantation has drawbacks of increased surgical trauma, long operation time, limited graft availability, and donor site morbidity; and allotransplantation may cause disease transmission and immune rejection [1].

In recent years, tissue engineering technology has provided new insights for the treatment of bone defects [2,3,4]. Dental stem cells as a cell source for tissue engineering, such as dental pulp stem cells (DPSCs), stem cells from human exfoliated deciduous teeth (SHEDs), and apical papilla stem cells (SCAPs), dental follicle progenitor cells (DFPCs) and periodontal ligament stem cells (PDLSCs) are the most widely derived [5,6]. Among them, Periodontal ligament stem cells (PDLSCs) are ideal seed cells for use in bone tissue engineering because they have many advantages over other stem cells, for example, abundant tissue availability, ease of access, and powerful tissue regenerative properties. However, stem cell-based therapies have their disadvantages. More than 99% of the implanted stem cells are trapped in the spleen, lung, and liver, while those cells close to the target tissues have a short life span and are prone to thrombosis, fever, and tumors [7,8,9,10,11]. Although this use of stem cells in combination with various scaffolds has long solved this problem, stem cells are still prone to cause oxidative stress and immune responses [12,13]. Nevertheless, recent studies suggest that the paracrine products of stem cells can be considered as an alternative to cell-based therapy as a cell-free therapy. Exosomes, a type of extracellular vesicle, typically have many properties from their source cells, such as amelioration of ischemic injury, promotion of tissue regeneration, nerve protection, nerve regeneration, and immune regulation, etc. [14,15,16,17]. Meanwhile, exosomes have the characteristics of low immunogenicity and do not elicit an immune response. Moreover, exosomes do not have the ability of self-replication, thus not leading to tumor formation. Compared with traditional stem cell therapy, such as direct transplantation and intravenous infusion, the application of exosomes is safer and more efficient [17,18].

Despite the many benefits of using exosomes, there are still some problems with delivering therapeutic doses of exosomes. They can be easily removed from circulation and may even accumulate in the liver, spleen, lungs, and gastrointestinal tract. Therefore, the delivery of exosomes requires a more efficient way to circumvent these risks [19]. Currently, the need for biocompatible, bioactive, and biodegradable materials for exosome delivery has attracted the attention of biomedical science to porous hydrogels [20,21,22,23].

Alginate is a sodium salt of alginic acid, as a naturally occurring polymeric material, and has exhibited excellent compatibility for biomedical purposes. Alginate hydrogels can be easily fabricated by crosslinking sodium alginate (SA) with calcium cations [24]. However, due to the poor mechanical properties of sodium alginate hydrogels, it is necessary to add other macromolecular biological materials to prepare composite hydrogels. Gelatin is a natural protein with amino acid sequences, it also serves as a natural polymer material with excellent biodegradability and biocompatibility profile. Gelatin blended with other biomaterials such as calcium phosphate ceramics, alginate, and chitosan has improved mechanical properties in scaffolding. Thus, we synthesized alginate-gelatin crosslinked (ADA-GEL) hydrogel to circumvent these pitfalls and limitations [25,26,27].

In this study, exosomes were isolated from PDLSCs, and they were combined with the ALG-GEL hydrogel for the first time to study its ability to promote bone regeneration in a rat alveolar bone defect model.

## 2. Materials and Methods

### 2.1. Isolation of Human Periodontal Ligament Stem Cells

We isolated human PDLSCs from periodontal ligaments of premolars or third molars extracted for orthodontic treatment purposes from young donors. This study protocol was approved by the Research Ethics Committee of Stomatological Hospital, affiliated with China Medical University (#21), and informed consent was obtained from all volunteers. PDLSCs were isolated from healthy PDL tissues of premolars and third molars extracted from 15 patients aged 12–18 years undergoing orthodontic treatment. Periodontal ligament tissues were minced and digested in a collagenase I solution at 37 °C for 1.5 h and then centrifuged. After centrifugation, the supernatant was discarded, and the precipitation was suspended in the α-MEM (Hyclone, Australia) containing 15% fetal bovine serum (FBS) (Clark, Australia) with l-glutamine (Invitrogen, USA) and antibiotics (Proteintech, Wuhan, China). Suspensions were inoculated in a T25 cell culture bottle at 37 °C in 5% CO_2_. After the cells grew out from around the tissue block, half of the culture medium was refreshed. After reaching 80% to 90% confluence, cells were detached by 0.25% trypsin-EDTA (Gibco, USA) and passaged.

### 2.2. Surface Antigen Analysis and In Vitro Multipotent Differentiation of PDLSCs

#### 2.2.1. Flow Cytometric Analysis

Surface antigens of PDLSCs were analyzed by flow cytometry. Briefly, after digestion, PDLSCs were centrifuged, washed with PBS, and then re-suspended in PBS containing 1% BSA (Sigma, Germany). After cell counting, a single-cell suspension was prepared. Then cells were incubated with an antibody conjugated to either allophycocyanin (APC) or phycoerythrin (PE) for half an hour on ice away from light. After being washed twice with PBS with 1% BSA, the cells were centrifuged and fixed. Finally, these cells were analyzed on the flow cytometer. Antibodies against CD34 (Beckman Coulter, USA), CD4 5(eBiosience, USA), CD44 (eBiosience, USA), and CD105 (BD Bioscience, USA) were used.

#### 2.2.2. Osteogenic Differentiation

PDLSCs were cultured in an osteogenic medium (OM), which consists of α-MEM with 10% FBS, 10 mM β-glycerophosphate, 50 mg/L ascorbic acid, 10 nM dexamethasone (Meilunbio, Dalian, China), 1% antibiotics and 2 mM l-glutamine for 14 days. Alizarin red staining (Solarbio, Wuxi, China) was used to detect the cellular mineralization hydrolysate.

#### 2.2.3. Adipogenic Differentiation

PDLSCs were cultured in an adipogenic medium which consists of α-MEM with 10% FBS, 100 nM dexamethasone, 0.2 mM indomethacin, 10 μM insulin, and 0.5 mM isobutylmethylxanthine (Meilunbio, Dalian, China) 1% antibiotics and 2 mM l-glutamine for 21 days. Oil red O staining (Solarbio, Beijing, China) was performed to detect the cell lipid drop level.

### 2.3. Isolation and Identification of Periodontal Ligament Stem Cells Derived Exosomes

PDLSCs were inoculated in a 10 cm culture dish. When the cells grew to 80% density, they were washed with PBS and replaced with α-MEM without FBS. After 48 h, 180 mL supernatant was collected and centrifuged at 300× *g* for 10 min, 2000× *g* for 10 min, and 10,000× *g* for 30 min to eliminate dead cells and cellular debris. Subsequently, these samples were filtered through a 0.22 μm filter and centrifuged at 100,000× *g* for 70 min. After the supernatant was removed, pellets were resuspended in PBS and centrifuged again at 100,000× *g* for 70 min [28]. Exosome pellets were resuspended in PBS and frozen at −80 °C for subsequent use.

The morphology of the exosomes was detected by transmission electron microscopy (TEM) (FEI, Netherlands). A BCA Protein Assay Kit (Beyotime, Shanghai, China) was used to quantify exosome protein concentrations. Markers of exosomes, including CD9 (Proteintech, Wuhan, China) and TSG101 (Proteintech, Wuhan, China), were assessed by western blot. The exosomes were analyzed for particle size distribution by TRPS analysis (qNano, Izon Science, New Zealand).

### 2.4. Exosomes Uptake Assay

Exosomes were labeled with DiD (red) dye (Meilunbio, Dalian, China) according to the manufacturer’s protocol. BMSCs were acquired from Cyagen Biosciences Inc. Then, BMSCs were incubated with the DiD-labeled exosomes for 24 h. The BMSCs were subsequently fixed, and the nuclei were stained with DAPI (Boster, Wuhan, China). The results were obtained using a Nikon laser scanning confocal microscopy (LSCM, Japan).

### 2.5. Cell Proliferation Assay and Cell Differentiation

The BMSCs were digested, re-suspended, and inoculated into a 96-well plate at a density of 3000 per well. Cells were treated for 1, 2, 3, 4 and 5 days with α-MEM containing 0, 10, and 20 μg/mL exosomes. The Cell Proliferation Experiment CCK-8 kit (Beyotime, Shanghai, China) was used to assess cell proliferation according to the manufacturer’s protocol.

Taking into account both yield and costs, 20 μg/mL exosomes were used for osteogenic induction. BMSCs were cultured in the PM (α-MEM containing 10% FBS and 1% antibiotics) or OM (α-MEM containing 10% FBS, 10 mM β-glycerophosphate, 50 mg/L ascorbic acid, 10 nM dexamethasone, 1% antibiotics) without exosomes or OM with exosomes. The alkaline phosphatase (ALP) activity was assessed by the ALP staining (Beyotime, Shanghai, China) after 14 days of induction, according to the manufacturer’s protocols.

### 2.6. Preparation and Characterization of Gelatin-Sodium Alginate Hydrogels

#### 2.6.1. Preparation of Gelatin-Sodium Alginate Hydrogel (Gel-Alg Hydrogel)

First, 0.6 g of gelatin (Meilunbio, Dalian, China) and 0.3 g of sodium alginate (Solarbio, Beijing, China) were weighed and added into 10 mL of sterile calcium-free PBS respectively, and fully dissolved at 70 °C. The gelatin-sodium alginate hydrogel was prepared by mixing the above solutions in a 1:1 (*V*:*V*) ratio. The final concentrations of gelatin and sodium alginate were 3% (*w*/*v*) and 1.5% (*w*/*v*). The gel solution was placed in a mold and in a 4 °C refrigerator. After molding, the gelatin-sodium alginate hydrogel was cross-linked by a certain amount of sterilized (2% *w*/*v*) CaCl_2_ solution (Aobox, Beijing, China). After this, CaCl_2_ was removed, and Gel-Alg hydrogel was washed in sterile PBS.

The hydrogel was freeze-dried in the freeze-dryer (Songyuan, Beijing, China) for 24 h, and then cut into small pieces. The surface of the sample was gilded by an ion sputter (Hitachi, Tokyo, Japan). The micromorphology of the freeze-dried gelatin-alginate hydrogel was observed by scanning electron microscopy (SEM) (Hitachi) under a 15 kV accelerating voltage.

#### 2.6.2. Cytotoxicity Test of the Gel-Alg Hydrogel

Preparation of leaching solution according to international standard ISO 10993. The Gel-Alg hydrogel was cleaned with sterilized PBS and then placed in a 12-well plate, followed by the α-MEM medium containing 12% FBS and 1% antibiotics in 37 °C for 24 h, collect the leaching solution and centrifuge at low speed. After that, the supernatant was filtered for sterilization as the leaching solution. Then BMSCs were inoculated in a 96-well plate with PM, and the inoculation density was 5000/well at 37 °C at 5% CO_2_. Until BMSCs adhered to the wall, PM was changed into a leaching solution. Then BMSCs were cultured for 24 h and 48 h. BMSCs were cultured in the α-MEM medium containing 12% FBS for the same time as the negative control group. CCK-8 assay was used for the cytotoxicity test, and the test was carried out according to the instructions.

### 2.7. Preparation and Property Determination of Exosome Composite Hydrogels

Gel-Alg hydrogel solution preparation as described above and the concentration of gelatine and sodium alginate were 6% (*w*/*v*) and 3% (*w*/*v*), respectively. Until the solution had cooled to 37 °C, a certain amount of hydrogel solution and the same amount of 2 μg/μL exosome suspension were plaed into a sterile mold and stirred gently to to ensure that they were evenly mixed. The final concentrations of gelatin and sodium alginate were 3% (*w*/*v*) and 1.5% (*w*/*v*), respectively. LSCM (Nikon, Tokyo, Japan) was used to observe exosomes labeled with DiD dye within the hydrogel to investigate their presence. A hydrogel without exosomes was used as a negative control.

### 2.8. In Vivo Experiment of Periodontal Membrane Stem Cell Exosomes Combined with Hydrogel to Promote Repairment of Alveolar Bone Defect

#### 2.8.1. Establishment of Alveolar Bone Defect Model

The animal experiments were sanctioned by the China Medicine University, and procedures were based on institutional guidelines.

A total of nine male SD rats (specific-pathogen-free) (SPF) (Sibeifu, Beijing, China), weighing 250–300 g, were selected. Preparation of the periodontal defect was as previously described [29,30,31]. The alveolar bone defect of about 2 × 1 × 0.8 mm^3^ was created in the mesial alveolar bone of the bilateral maxillary first molar mesial root. The SD rats were randomly separated into three groups: (1) PBS was administered to the control group (control, *n* = 6); (2) a group received treatment with Gel-Alg hydrogel (hydrogel, *n* = 6); and (3) a group treated with hydrogel combined with exosomes (hydrogel + exosomes, *n* = 6). (*n*: the number of alveolar bone defects in each group). After surgery, antibiotics were injected intramuscularly to prevent infection. Four weeks after the surgery, the rats were killed via anesthetic overdose and then decapitated immediately. The maxillary with the defects was obtained and fixed with 4% paraformaldehyde.

#### 2.8.2. Micro-CT Scan Analysis

After fixation with 4% paraformaldehyde, the maxilla with the bone defect was scanned by micro-CT (Bruker, Belgium). After scanning, the system’s software was used to reconstruct the ROI and analyze the results.

#### 2.8.3. Histological Analysis

Following micro-CT analysis, the maxillas were decalcified with 10% ethylenediaminetetraacetic acid disodium (EDTA), and then samples were embedded in paraffin after being dehydrated in a gradient series of alcohol. Subsequently, these samples were sliced into 5 μm-thick slices. Then, for histological analysis, the sections were subjected to hematoxylin and eosin (HE) staining and Masson staining to visualize defect healing and bone formation.

### 2.9. Statistical Analysis

SPSS 23.0 software (Chicago, IL, USA) was used for data analysis. The mean and standard deviation (SD) were used to present all data. The differences between groups were analyzed by one-way ANOVA and Student’s t-test analyses. *p* < 0.05 was considered statistically significant.

## 3. Results

### 3.1. Separation and Identification of hPDLSCs

**Figure 1:**
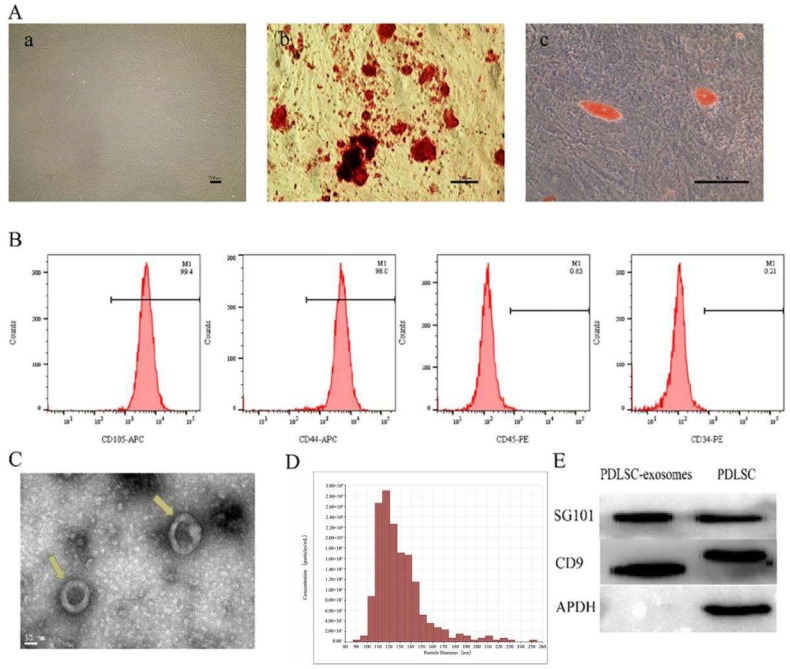
Characterization of PDLSCs and PDLSCs-derived exosomes. (**A**) Characterization of PDLSCs (**a**) Representative images of PDLSCs. Spindle shape with vortex distribution (**b**) Representative images of osteogenesis of PDLSCs stained with Alizarin red staining. A large number of red mineralized nodules were observed. (**c**) Representative images of adipogenesis of PDLSCs stained with Oil Red O. Red lipid droplets were formed. (**B**) Flow cytometric analysis of surface markers CD105, CD44, CD45, CD34 in PDLSCs. CD105 and CD44 are highly expressed, while CD45 and CD34 are low expressed. (**C**) Exosomes morphology of a cup-shape or circle observed by TEM (indicated by the yellow arrowheads). (**D**) Particle size distribution of exosomes concentrated between 90 and 150 nm detected by TRPS. (**E**) Western blot analysis of the exosomal markers TSG101 and CD9.

We have successfully isolated PDLSCs from human PDL, and the cells exhibited the morphology of a uniform spindle-shaped (Figure 1(Aa)). The results of differentiation experiments demonstrated that PDLSCs are capable of osteogenic and adipogenic differentiation in vitro (Figure 1(Ab,Ac)). Flow cytometry analysis showed that PDLSCs were positive for MSCs markers, including CD105 and CD44, and were negative for hematopoietic stem cell markers, CD34 and CD45 (Figure 1B).

### 3.2. Characterization of PDLSCs-Exos

Transmission electron microscopy (TEM) (Figure 1C) and Tunable resistive pulse sensing (TRPS) (Figure 1D) analysis demonstrated that the exosomes appeared to have the morphology of a cup-shape or circle, and the exosome diameter concentrated between 90 and 150 nm. Western blotting assays revealed the exosomal markers CD9 and TSG101 (Figure 1E). These results confirmed that we have successfully extracted exosomes.

### 3.3. Ingestion of PDLSCs-Exos by BMSCs

**Figure 2:**
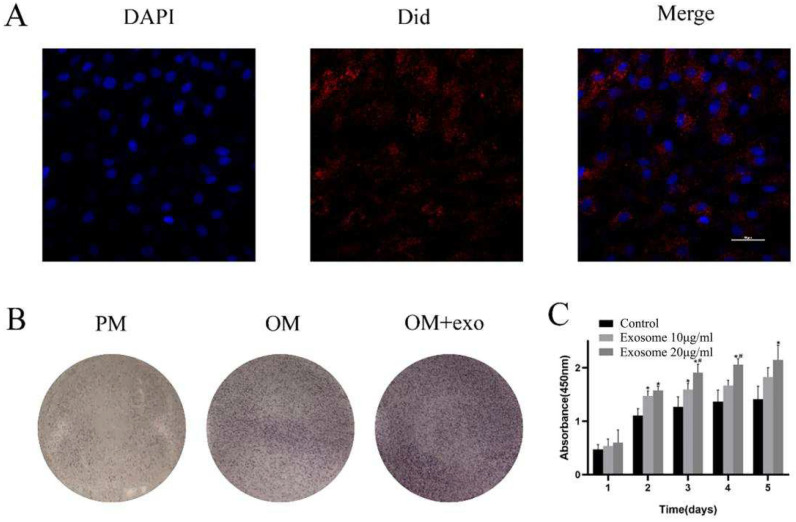
Effects of exosomes on proliferation and osteogenic differentiation of BMSCs (**A**) Ingestion of exosomes by BMSCs (**B**) ALP staining after 14 days of osteogenic induction (**C**) CCK-8 assay results; Asterisk (*), *p* < 0.05 compared to the control group; pound sign (#), *p* < 0.05 compared to the exosome 10 μg/mL group.

Images of exosome uptake by BMSCs were visualized using LSCM (Figure 2A). The image showed that DiD-labelled PDLSCs-Exos (red dots) were internalized by the cells and distributed around the nucleus.

### 3.4. Effects of PDLSCs-Exos on Proliferation and Osteogenic Differentiation of BMSCs

The CCK-8 assay was used for the examination of cell proliferation. The OD values revealed that the proliferation of BMSCs can be significantly enhanced by 10 μg/mL and 20 μg/mL PDLSCs-Exos; the BMSCs treated with 20 μg/mL exosomes showed a greater capacity for proliferation compared with that treated with 10 μg/mL (Figure 2B).

ALP staining after osteogenic induction of BMSCs for 14 days in the OM with PDLSCs-Exos was observably increased in comparison with that of BMSCs in PM and OM without PDLSC-Exos (Figure 2C).

### 3.5. Characterization of the Gelatin-Alginate Hydrogel

**Figure 3:**
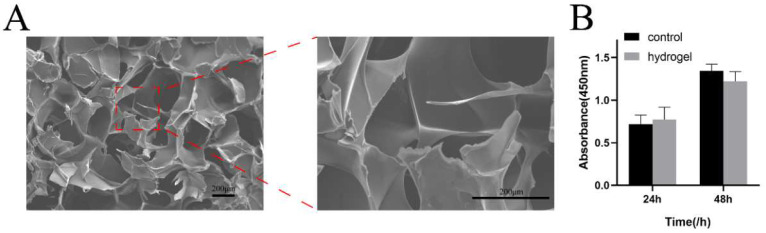
Characterization of hydrogels (**A**) SEM image of hydrogel scaffold (**B**) CCK-8 assay results of cytotoxicity test.

SEM micrographs of the hydrogel samples showed porous and interconnected structures. Furthermore, the pore wall’s surface was smooth (Figure 3A). The CCK-8 assay was used for cytotoxicity evaluation, as shown in Figure 3B. After 24 h, the survival rate of BMSCs in the hydrogel group was not significantly different from that of the negative control group. Furthermore, from 24 h to 48 h, OD values showed obvious proliferation of BMSCs, indicating that cells cultured in the leaching solution can grow normally, proving that gelatin-alginate hydrogel has no obvious cytotoxicity and can be used as a hydrogel scaffold for future studies.

### 3.6. Exosomes Detection from Hydrogels

**Figure 4:**
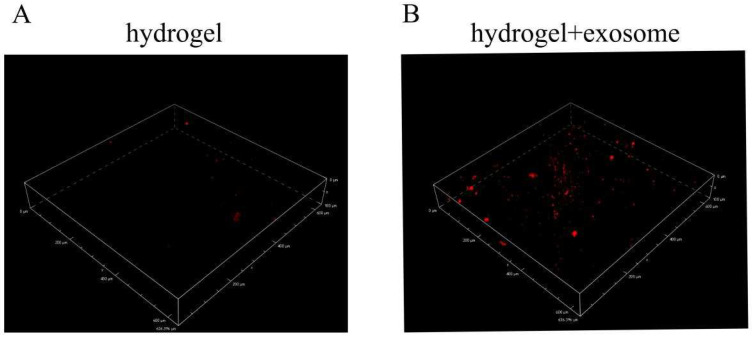
LSCM images of (**A**) hydrogel (control) and (**B**) hydrogel with red fluorescence DiD-labeled exosomes (hydrogel + exosome).

LSCM image of the hydrogel with PDLSCs-Exos can be seen in Figure 4B, When compared to the control hydrogel (Figure 4A), a large number of exosomes labeled with red fluorescent DiD dye were distributed in the hydrogel material (Figure 4B).

### 3.7. Micro-CT Results of Bone Regeneration

**Figure 5:**
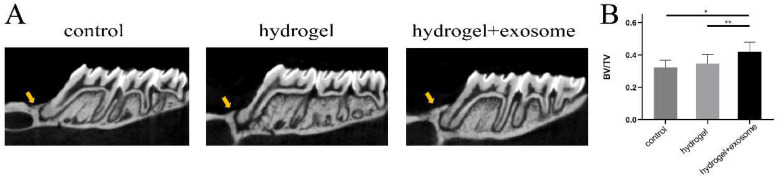
Micro-CT assessment of bone formation at 4 weeks post-surgery (**A**) representative micro-CT images for the different groups at the defect site (indicated by the yellow arrowheads) (**B**) BV/TV; (*), *p* < 0.05 compared to the control group; (**), *p* < 0.05 compared to the hydrogel group.

Figure 5A shows representative micro-CT views. The results reveal that both the control and hydrogel groups reported fewer new bone regeneration. More new bone was discovered in the hydrogel + exosome group. Similarly, the BV/TV results showed that the hydrogel + exosome group formed more new bone than the other groups (Figure 5B).

### 3.8. Histological Analysis of Bone Regeneration

**Figure 6:**
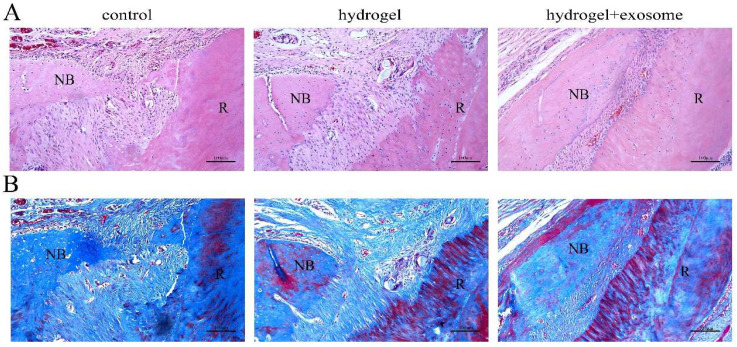
Histological evaluations of bone formation at 4 weeks post-surgery (**A**) HE staining images of alveolar bone in control, hydrogel and hydrogel+exosome groups, respectively. Compared with the control and hydrogel groups, the hydrogel+exosome group had more new bone deposition. (**B**) Masson staining images of alveolar bone in control, hydrogel and hydrogel+exosome groups, respectively. Compared with the control and hydrogel groups, the hydrogel+exosome group had more new bone deposition. R: root; NB: new bone of periodontal regeneration.

The HE staining results can be seen in Figure 6A. In comparison with the hydrogel group and control group, the hydrogel + exosome group had more new bone deposition. Meanwhile, the Masson staining demonstrated that more bone-like tissue formation was detected in the hydrogel + exosome group than in the other two groups (Figure 6B).

## 4. Discussion

The repair of alveolar bone defects remains a clinically significant problem [32]. Stem cell therapy has proved to be an effective treatment for bone repair [15]. However, several studies have provided that the therapeutic effects of MSCs are mainly through paracrine mechanisms. The exosome is considered to be an eventful paracrine factor in MSCs and could be applied for tissue regeneration.

Periodontal Ligament Stem Cells (PDLSCs), a type of dental stem cell, are a population of MSCs isolated from the periodontal ligament (PDL). They are capable of self-renewal and multilineage differentiation into cell types such as odontoblasts/osteoblasts, adipocytes, and neuron-like cells. When compared to MSCs, PDLSCs are more widely available, have fewer ethical concerns, and are less expensive [33]. These advantages make it a suitable applicant for bone repair. Increasing evidence has demonstrated that exosomes from stem cells of dental origin can promote tissue regeneration [34]. However, no previous research has been done on the effect of PDLSCs-Exos on bone defect repair. As a result, we hypothesized that PDLSCs-Exos might be a promising strategy for repairing alveolar bone defects in this study.

BMSCs have incubated with fluorescence dye-labeled exosomes for 24 h and then observed by LSCM. These results suggested that exosomes can be uptaken by BMSCs. Then, we assessed the effect of different concentrations of exosomes on the proliferation and osteogenic differentiation capacity in BMSCs. The effect of exosomes on osteogenic differentiation in vitro was confirmed by ALP staining. The results found that exosomes from PDLSCs could promote the proliferation and osteogenic differentiation of BMSCs in vitro.

To assess the ability of exosomes to promote the repair of alveolar bone defects in vivo, we applied a hydrogel as a scaffold. Alginates are being used in various biomedical applications such as hydrogels and scaffolds. We use CaCl_2_ cross-linked to form the hydrogel. The hydrogel should be rinsed at least 10 times to remove the excess CaCl_2_. The SEM microscopic structure showed a porous and multi-layered structure of gelatin-alginate hydrogel, which favors living cell growth and water uptake. Meanwhile, the image of LSCM showed that the DiD-labeled PDLSC-Exos were embedded in the hydrogel. All these properties clearly showed that the hydrogel was fit to deliver exosomes to further prevent rapid clearance in the circulation.

Exosomes have been shown to promote bone defect repair in previous studies, and various methods have been explored to combine biomaterials with exosomes to prolong the duration of exosomes and thus improve the effect of exosomes. Chew et al. [29] combined exosomes derived from BMSCs with a sponge to repair alveolar bone defects in rats. Wu et al. [35] incorporated β-TCP with exosomes derived from deciduous tooth stem cells for alveolar bone defect repairment in rats. Good results were obtained in these studies. In our study, PDLSCs-Exos were combined with hydrogel for the first time to explore the osteogenic properties of PDLSCs-Exos in vitro and in vivo. Based on the results of animal experiments, we found that the hydrogel + exosome group had the greatest bone regeneration in the alveolar bone defects at 4 weeks post-surgery compared with other groups.

However, this study has some limitations. The use of lipophilic dye (DiD) to label exosomes in exosome uptake experiments and detection of exosomes in hydrogels was not ideal. This lipophilic dye can also bind to the membrane fragments in fragmentary exosomes and also show a stronger fluorescence intensity. Therefore, this fluorescent dye cannot fully reflect the integrity and biological activity of exosomes [36,37]. Furthermore, although our study revealed a beneficial effect of exosomes with hydrogel on bone regeneration, we did not compare it with the osteogenic effect of stem cells with hydrogel. Therefore, more studies are needed to explore these problems.

## 5. Conclusions

In our study, PDLSCs and PDLSCs-derived exosomes were successfully isolated and PDLSCs-Exos were combined with Gel-Alg hydrogel for the repair of alveolar bone defects in SD rats at the initial stage. The results demonstrated that the hydrogel successfully delivered PDLSCs-Exos for repairing alveolar bone defects in rats in vivo.

## Data Availability

The data presented in this study are available in the paper.
